# Post-acute COVID-19 outcomes in children requiring hospitalisation

**DOI:** 10.1038/s41598-022-12415-x

**Published:** 2022-05-17

**Authors:** Cara J. Bossley, Ema Kavaliunaite, Katharine Harman, James Cook, Gary Ruiz, Atul Gupta

**Affiliations:** Department of Paediatric Respiratory Medicine, King’s College Hospital NHS Foundation Trust, London, SE5 9RS UK

**Keywords:** Diseases, Medical research

## Abstract

Post-acute COVID-19 causes long term sequalae in adults. This is less well described in children. We performed clinical assessments on a large cohort of children and young people admitted with a positive SARS-CoV-2 RNA swab. We assessed for symptoms of post-acute COVID-19 syndrome after 4 weeks or more. We found that most (85%) of children made a full recovery following SARS-CoV-2 infection. A small number had symptoms which lasted for more than 4 weeks, most of which had resolved at 3 months. Symptoms included dry cough, fatigue and headache. One patient suffered from anosmia. We conclude that most children and young people do not suffer from past-acute COVID-19 syndrome, and make a full recovery from infection.

## Introduction

Most of our current insights into the long term sequelae of COVID-19 come from adults who have recovered from symptomatic acute SARS-CoV-2 infection often with multiple organ involvement^1^. Children rarely have this typical acute presentation. However, symptomatic acute infection may not be a necessary precursor of the late sequelae of SARS-CoV-2 infection^2^. Post-acute COVID-19 has been defined as symptoms persisting or sequelae developing 4 weeks beyond the initial infection^2^. Recent data from the office of National Statistics in the UK suggest late symptoms occur in children and young people (CYP). We previously reported on children admitted to our hospital with SARS-CoV-2 RNA positivity during the first and second COVID-19 waves in in the UK^3,4^. We aimed to explore whether any of these children might have late symptoms in common with post-acute COVID-19 syndrome in adults^1^.

## Methods

The subjects were CYP up to 18 years of age with SARS-CoV-2 RNA positivity who were admitted between 1 March 2020 and 19 January 2021 to King’s College Hospital^3,4^. None of the children included had PIMS-TS.

We assessed symptoms associated with post-acute COVID-19 syndrome, defined as symptoms persisting for 4 weeks or longer^1^. The CYP were aged 18 years or younger admitted between 1 March 2020 and 19 January 2021 to King’s College Hospital with positive SARS-CoV-2 RNA positivity.

The assessment was performed using a standardised clinical proforma (Appendix 1) over the telephone. The telephone review was made by Paediatric Respiratory consultants in March 2021 and therefore from 3–12 months after the admission.

Methods were performed in accordance to the guidelines and regulations of the trust.

This was a prospective observational cohort study. We entered the results of the assessments into an excel database, and analysed the difference between those with and without longer lasting symptoms and the severity of their initial presentation using the Chi squared test or fishers exact test. A significance of p < 0.05 was deemed significant.

### Ethics approval

Approved from the ethics committee of King’s College as a service evaluation project. Methods and protocols were approved by King’s College audit committee as a clinical audit. Parents/guardian’s of children gave full informed consent to perform the assessment at the time of being assessed.

## Results

A total of 88 CYP were admitted with a range of conditions. 30 (34%) were infants, 15 (17%) were of preschool, 12 (13.5%) of primary and 31 (35.5%) of secondary school age. 54 (61%) were male, 43 (49%) had an underlying co-morbidity. Acute disease severity was classified according to modified World Health Organisation classifications^2^. 24/88 (27%) were asymptomatic or had incidental findings and 42/88 (48%) mild, 11/88 (12.5%) moderate, 5/88 (5.5%) severe and 6/88 (7%) critical disease. We were unable to contact 17/88 (19%) at the time of the survey.

There were 71 patients available for follow up, 42/71 (59%) were male and the mean age 6.7 years (range; 11 days–17 years). Most (60/71, 85%) patients had made a complete recovery. A small proportion, namely 11/71 (15%) had symptoms beyond 4 weeks of discharge (Tables 1 and 2). These symptoms had resolved in 1–3 months in 5/11 (45%) (Table 1). Four of these 5 patients had an acute respiratory presentation and the fifth had a history of asthma making it difficult to attribute any persistence of symptoms confidently to COVID-19. Most of the more prolonged symptoms in the other patients were similarly unlikely to be due to COVID-19 and some of these patients were under investigation for a specific cause.

**Table 1:** Children and young people with symptoms beyond 4 weeks, which had resolved.

Child	Modified WHO severity	Age and sex	Co-morbidity	Presentation	Length of symptom	Long term effect	Related to COVID
1	Mild	11 days Male	None	Fever Cough	3 months	Cough Episode of post-tussive vomiting	Yes
2	Moderate	5 months Male	None	Fever Shortness of breath	4 months	Shortness of breath Noisy breathing Consolidation on chest X-ray	Possible
3	Mild	8 months Male	None	Fever	6 months	Low neutrophils	Possible
4	Mild	10 months Female	None	Barking cough and vomiting	4 weeks	Cough	Possible
5	Severe	14 months Male	None	Wheeze	8 weeks	Cough	Possible
6	Incidental findings	13 year Male	Oesophagitis Constipation Obesity ADHD	Asymptomatic	11 months	Cough Occasional fever	Possible
7	Moderate	17 year Male	Asthma	SOB Diarrhoea and vomiting	6 weeks	Shortness of breath Lack of energy Fatigue	Possible
8	Mild	17 year Male	Asthma Obesity Pancreatitis	Abdo pain	2 months	Colitis/diarrhoea Joint pain Weight loss	Possible
9	Incidental findings	13 year Female	Obesity	Appendicitis causing sepsis, prolonged PICU admission	6 months	Knee pain Elbow pain Mild shortness of breath Poor appetite	Possible

**Table 2:** Children and young people with ongoing symptoms.

Child	Modified WHO severity	Age and sex	Co-morbidity	Presentation	On going symptoms/issues	Impact of on-going symptoms	Related to COVID?
1	Mild	11 year Male	None	Abdominal pain did have appendicectomy	Joint pain Fatigue Headaches	Returned to school but doing less sports than previously	Possible
2	Severe	13 year Male	Duplication of chrom 6 (6p21) IgA deficiency Ehlers Danlos syndrome Gastro-oesphageal reflux	Fever Dry cough Poor appetite	Anosmia Shortness of breath Cough Headache Fatigue Confusion Sleep disturbance Weakness	Ongoing tiredness and fatigue	Probable

There was no association between severity of presentation and probability of more persistent symptoms. The most common prolonged symptom was a dry cough but it was seen in only 5/71 (7%) patients. This has been well described in both paediatric and adult cohorts^5,6^. Shortness of breath was seen in 4/71 (6%), fatigue in 3/71(4%) and headaches in 2/71 (3%). One patient (1.4%) suffered from anosmia. None of the 11 symptomatic patients had any symptoms that limited their daily activities like eating, drinking or getting dressed. All the children who were at school or nursery before the hospitalisation, had returned to education.

## Discussion

We report the longer term effects of children hospitalised with COVID-19 infection. We have demonstrated that most children make a full recovery from the infection and do not suffer from post-acute COVID-19. A small proportion of children had on-going symptoms post 4 weeks of discharge but then recovered within 3 months. These results are reassuring for children, and underline the fact that children are less likely to be adversely affected by COVID-19 infection.

There was a very small proportion of children who had on going effects, but it is difficult to know if some of the symptoms are directly attributable to the COVID-19 infection, and some effects may be incidental. We do report a post COVID cough, and this seemed to be a consistent finding, however effected only a small proportion of patients. This has been described in adult post-acute COVID infection^5^ and more recently in children^6^. In our cohort of patients most children did recover from this within 3 months. This cough was the main symptom in the majority of the patients and this could be a post-infectious hyper-reactive airway syndrome seen in children following other viral infections. Interestingly, a prospective single centre study performed in Israel, showed that children seen in a designated multi-disciplinary clinic for long COVID revealed impairment in lung function testing in 45% of patients with cardiorespiratory symptoms, however there was not a control group, and the abnormalities comprised of mild obstructive patterns, with more than half exhibiting bronchodilator reversibility^7^.

Our findings are in contrast to a review of Dutch patients, whereby a large number of children were identified as having long COVID^8^, however, in contrast to our report this study included patients with suspected COVID, and not all patients had a positive PCR test. Our cohort differs as all of our children were in hospital at the time of testing positive for SARS-CoV-2. There have been further studies which have showed possibility of long COVID. A recent narrative review of 14 paediatric studies, it was highlighted that many of the studies included patients who did not necessarily have a positive PCR test, rather self reported COVID-19 infection, and thus could include problems from other viruses^9^. The review found only 4 studies with control groups. They found that there were major limitations in all of the studies, which probably accounts for the wide prevalence of COVID 19 between the studies of between 4 to 66%^9^. The review concluded the evidence for long COVID in CYP to be limited, with the absence of a control group meaning that it is difficult to differentiate symptoms attributable to COVID-19 vs those related to the pandemic in general. The review did however feel further studies in this group are important, to help guide us as to whether immunisation in this group could be necessary to prevent long COVID.

This follow up study of a cohort of CYP hospitalised with COVID-19, and demonstrates that the majority of CYP (85%) did not encounter any long-term sequelae. Assuming this data can be generalised globally, it would help to understand the natural history of this virus in CYP and enable prioritisation of follow-up care.

## Conclusion

We conclude from our assessments, that most children admitted with COIVD-19 make a full recovery. There are very small proportion of children who had longer lasting effects but these could be effects seen commonly following other viral illnesses.

## Supplementary Information

Supplementary Information.

## Data Availability

We have available data as needed. The datasets used and/or analysed during the current study are available from the corresponding author on reasonable request. All the patients with prolonged symptoms are described in the tables.
