# Supplementary material for: Post-acute COVID-19 outcomes in children requiring hospitalisation
Source: Sci Rep. 2022 May 17;12:8208. doi: 10.1038/s41598-022-12415-x (PMC9113067; doi:10.1038/s41598-022-12415-x)
Supplement: Supplementary file 1 — Supplementary Information. [file 41598_2022_12415_MOESM1_ESM.docx]

**Appendix 1**

| **Acute COVID Follow up**  *We are getting in touch with children & young people who have been discharged after having had a positive test for coronavirus disease (COVID-19). The purpose of this call is to find out if your child are experiencing problems related to your recent illness with coronavirus. We will document this in your clinical notes. We will use this information to direct you to services you may need and inform the development of these services in the future.* | |
| --- | --- |
| Date of Positive swab the infection (admission date) |  |
| Date of Onset of symptoms |  |
| Date of discharge |  |
| Date of the call / follow up |  |
| Hospital number |  |
| Age |  |
| Sex |  |
| Acute Disease severity (Modified WHO) |  |
|  | |
| Has your child made a full recovery or are they still troubled by symptoms? | Yes / No |
| If still troubled has your child had any of the following symptoms 4 weeks after discharge from the hospital or any pre-existing symptoms got worse? | |
| Fever | Yes / No |
|  |  |
| Any joint pain | Yes / No |
| Diarrhoea | Yes / No |
| Nausea/vomiting | Yes / No |
| Abdominal pain | Yes / No |
| Loss of appetite or weight | Yes / No |
| Anosmia (‘no sense of smell’ | Yes / No |
| Sense of taste | Yes / No |
| Do you feel more breathless than you were pre your COVID illness | Yes / No |
| Do you have a cough (different from any cough you may have had before COVID-19) | Yes/ No |
| Chest pain | Yes / No |
| Palpitations? (sense that you can feel your heart pounding or racing) | Yes / No |
|  |  |
| Headache | Yes / No |
| Vertigo / Dizziness | Yes / No |
| Memory loss / confusion | Yes / No |
| Do you find yourself feeling anxious/worrying more than you used to pre COVID illness? | Yes / No |
| Do you feel fatigued (worn out/lacking energy or zest) compared with how you were before your COVID illness? | Yes / No |
| How is your physical strength? Do you feel so weak that it still limiting what you can do (more than you were pre your COVID illness)? | Yes / No |
| Is your sleep disturbed (more than it was pre-COVID)? | Yes / No |
| How long have symptoms gone on for? | |
| Did you seek medical attention for symptoms? | |
| Did you/your child have symptoms for longer than 4 weeks after discharge that are now better?  If Yes, then complete symptom questions to identify what those symptoms were and also ask when those symptoms got better | |

**Decision guide:**

Any positive symptoms will need MDT discussions & further investigations / evaluation
